# Clinical Value of Total Intravenous Anesthesia with Sufentanil and Propofol in Radical Mastectomy

**DOI:** 10.1155/2022/7294358

**Published:** 2022-08-05

**Authors:** Lingyan Qu, Xiaoqing Wu

**Affiliations:** ^1^Department of Anesthesiology, Yantaishan Hospital, Yantai, 264000 Shandong, China; ^2^Department of Radiotherapy, Central Hospital Affiliated to Shandong First Medical University, Jinan, 250013 Shandong, China

## Abstract

**Objective:**

To investigate the clinical value of sufentanil combined with propofol for total intravenous anesthesia (TIVA) in radical mastectomy.

**Methods:**

The data of 120 patients undergoing radical mastectomy of breast cancer in our hospital from February 2020 to February 2021 were retrospectively analyzed, and they were randomly assigned to the experimental group (EXG) (*n* = 60) and the control group (COG) (*n* = 60). The anesthesia maintenance scheme was 0.01–0.03 *μ*g/(kg·min) of sufentanil + 80–100 *μ*g/(kg·min) of propofol in EXG and 3 *μ*g/(kg·h) of fentanyl + 80–100 *μ*g/(kg·min) of propofol in COG. The hemodynamic indices, stress indexes, postoperative pain scores, and incidence of adverse reactions were compared between EXG and COG.

**Results:**

The heart rates (HR) and mean arterial pressure (MAP) after tracheal intubation (T_2_) and at separation of deep tissues (T_3_), tracheal extubation (T_4_), and the end of surgery (T_5_) were markedly lower in EXG than in COG (*P* < 0.001). The stress indexes and postoperative pain scores at 1 h (T_6_), 6 h (T_7_), and 12 h (T_8_) after surgery were lower in EXG than in COG (*P* < 0.001). The incidence of dizziness, headache, pruritus, and emergence agitation in EXG was lower compared with that in COG (*P* < 0.05).

**Conclusion:**

Sufentanil combined with propofol for TIVA can stabilize intraoperative hemodynamic indices of patients undergoing radical mastectomy, alleviate perioperative stress response, and reduce pain perception. Therefore, this anesthesia method is safe and merits clinical promotion.

## 1. Introduction

Breast cancer (BC) is a type of cancer caused by the uncontrolled proliferation of mammary epithelial cells, and its incidence ranks first among female tumors worldwide [1], with 52.9% occurring in developing countries [2]. As the most populous developing country, China has the highest number of new BC patients in the world each year and the number of patients needing radical mastectomy increases yearly [3, 4]. Since radical mastectomy is a body surface surgery with significant trauma and has a major impact on the respiratory system and circulatory system of patients, there is a clinical need for highly effective anesthesia modalities with good analgesic effects, such as total intravenous anesthesia [5]. Total intravenous anesthesia (TIVA) refers to the combination of multiple intravenous anesthetics after intravenous anesthesia induction to maintain anesthesia by intravenous injection. Propofol is one of the most common TIVA drugs [6, 7], with significant advantages such as rapid onset, rapid postoperative recovery, complete recovery of various system functions, and a low incidence of adverse reactions such as nausea and vomiting. Moreover, its combined use with other anesthetics can further improve the analgesic function and enhance the anesthetic effects. At present, opioids are often combined with propofol in thoracic surgery and neurosurgery, such as fentanyl, sufentanil, and remifentanil [8], in which remifentanil has a strong inhibitory effect on the respiratory system and its comprehensive anesthesia effect is inferior to sufentanil [9]. Fentanyl is an early opioid analgesic with strong analgesic effects and can reduce postoperative delirium [10]. However, not much is known about the combined use of sufentanil and propofol for TIVA and the comparison results of effects between fentanyl and sufentanil remain unclear. Based on this, this study will explore the application effects of fentanyl combined with propofol and sufentanil combined with propofol for TIVA, trying to provide a reference for clinical practice.

## 2. Materials and Methods

### 2.1. Study Design

This retrospective study was conducted in our hospital from February 2020 to February 2021 to explore the clinical application value of sufentanil combined with propofol for TIVA in radical mastectomy of breast cancer.

### 2.2. General Information

The data of 120 female patients undergoing radical mastectomy in our hospital from February 2020 to February 2021 were retrospectively analyzed, and they were randomly assigned to the experimental group (EXG) (*n* = 60) and the control group (COG) (*n* = 60). The clinical data of both groups were shown in Table 1. No notable differences were found in the general data between EXG and COG (*P* > 0.05).

Inclusion criteria are as follows: (1) patients were diagnosed with BC by pathological examination [11], (2) patients had indications for radical mastectomy, (3) patients were treated in our hospital throughout the whole process and had complete clinical data, and (4) patients were aged ≥18 years old.

Exclusion criteria are as follows: (1) Patients who were unable to communicate with others due to factors such as hearing disorders, language disorders, unclear consciousness, or mental illness; (2) patients with dysfunctions in important organs such as the heart, lung, liver, and kidney; (3) patients allergic to drugs involved in the study; (4) patients with long-term administration of analgesics or sedatives; (5) patients with severe anemia; (6) patients with a history of acute and chronic respiratory diseases; and (7) those in pregnancy or lactation.

### 2.3. Moral Considerations

This study was in accordance with the principles of the Declaration of Helsinki (2013) [12]. Patients knew the purpose, significance, content, and confidentiality of the study and signed the informed consent.

### 2.4. Methods

All patients were routinely fasted and prohibited from drinking before surgery. After entering the operating room, the upper limb vein access was opened and 8–10 ml/(kg·min) of ringer lactate solution (Sichuan Kelun Pharmaceutical Co. Ltd.; NMPA approval no. H20055488) was intravenously dripped. ECG and blood oxygen saturation were routinely monitored during surgery, while the bispectral index (BIS) and muscle relaxation were also monitored. In EXG, 1–2 mg/kg of propofol (Shenyang First Pharmaceutical Co. Ltd. of Northeast Pharmaceutical Group; NMPA approval no. H20031358), 0.1–0.2 *μ*g/kg of sufentanil (Yichang Humanwell Pharmaceutical Co. Ltd.; NMPA approval no. H20054171), 0.3 mg/kg of midazolam (Jiangsu NHWA Pharmaceutical Co. Ltd.; NMPA approval no. H10980026), and 0.1 mg/kg of vecuronium bromide (Zhejiang Xianju Pharmaceutical Co. Ltd.; NMPA approval no. H19991172) were intravenously injected for anesthesia induction. When TOFT4/T1 = 0 was monitored by a muscle relaxation monitor, the direct vision orotracheal intubation was carried out. During surgery, 0.01–0.03 *μ*g/(kg·min) of sufentanil and 80–100 *μ*g/(kg·min) of propofol were used for anesthesia maintenance. The infusion of sufentanil was stopped at 30 min before the end of the surgery, and propofol was stopped at 10 min before the end of the surgery. The anesthesia method in COG was the same as that in EXG. In COG, 1–2 mg/kg of propofol, 2 *μ*g/kg of fentanyl (Yichang Humanwell Pharmaceutical Co. Ltd.; NMPA approval no. H42022076), 0.3 mg/kg of midazolam, and 0.1 mg/kg of vecuronium were intravenously injected for anesthesia induction. The anesthesia maintenance scheme was 3 *μ*g/(kg·h) of fentanyl combined with 80–100 *μ*g/(kg·min) of propofol. The infusion of fentanyl was stopped at 30 min before the end of the surgery, and propofol was stopped at 10 min before the end of the surgery.

Both groups received pump infusion of atracurium (Shanghai Hengrui Pharmaceutical Co. Ltd.; NMPA approval no. H20061298) to maintain muscle relaxation. The pumping was stopped at 30 min before the end of the surgery, and TOFT4/T1 < 15% and 40–60 BIS were maintained during surgery. After surgery, 0.3–0.6 mg/kg of neostigmine (Shanghai Zhongxi Sunve Pharmaceutical Co. Ltd.; NMPA approval no. H31020217) and 0.1–0.3 mg/kg of atropine (Tianjin Huajin Pharmaceutical Co. Ltd.; NMPA approval no. H12020417) were intravenously injected to antagonize residual muscle relaxants.

### 2.5. Observation Criteria

#### 2.5.1. Hemodynamic Indices

The noninvasive anesthesia depth detector (Sichuan Intelligent Electronics Industry Co. Ltd.; Sichuan Medical Products Administration Approval no. 20062210024) was connected with the monitoring electrode placed in the middle of the forehead and the left mastoid and the reference electrode placed in the left forehead. After wearing the headphones, auditory stimulation was performed at 70 dB and 6.9 Hz. The heart rate (HR) and mean arterial pressure (MAP) of both groups were observed before anesthesia (T_1_), after tracheal intubation (T_2_), and at separation of deep tissues (T3), tracheal extubation (T_4_), and the end of surgery (T_5_).

#### 2.5.2. Stress Indexes

The radial artery blood (5 ml) of patients was collected before anesthesia (T_1_), and at 1 h (T_6_), 6 h (T_7_), and 12 h (T8) after surgery. After centrifugation, the upper plasma was taken. The concentrations of epinephrine (E) and norepinephrine (NE) were determined by the modified fluorimetric method (Chuzhou Ruigu Biotechnology Co. Ltd.; Anhui Medical Products Administration Approval no. 20202400353), and the cortisol (COR) levels were determined by the chemiluminescence method (Beckman Coulter Co. Ltd. (America); NMPA (I) 20082403092).

#### 2.5.3. Postoperative Pain Scores

The verbal rating scale (VRS) [13] was used to evaluate the pain degree of both groups at 1 h (T_6_), 6 h (T_7_), and 12 h (T_8_) after surgery. VRS was composed of a group of adjectives to describe pain from the lightest to the heaviest grading 1–4 points. The higher the score, the more severe the pain.

#### 2.5.4. Incidence of Adverse Reactions

The types of adverse reactions in both groups were recorded and the incidence of adverse reactions was calculated. Adverse reactions referred to harmful reactions unrelated to the purpose of treatment in the course of prevention, diagnosis, or treatment of diseases by normal usage and dosage of drugs.

### 2.6. Statistical Treatment

The data were processed by Software SPSS20.0 and graphed by GraphPad Prism 7 (GraphPad Software, San Diego, USA). The data in the study comprised enumeration data and measurement data, tested by *X*^2^ and *t*-test. The differences were statistically significant at *P* < 0.05.

## 3. Results

### 3.1. Comparison of Hemodynamic Indexes

**Figure 1:**
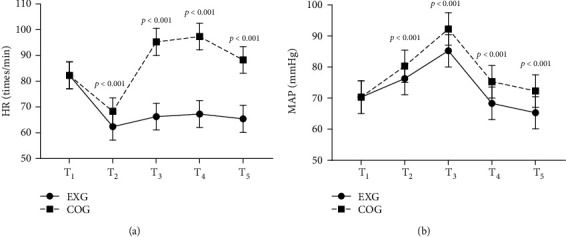
Comparison of hemodynamic indexes ($\bar{x}\pm \text{s}$). (a) showed the heart rate (HR) (times/min); (b) showed mean arterial pressure (MAP) (mmHg).

The heart rates (HRs) and mean arterial pressure (MAP) at T_2_, T_3_, T_4_, and T_5_ were markedly lower in EXG than in COG (*P* < 0.001), as presented in Figure 1.

No obvious differences were shown in HR at T_1_ between EXG and COG (82.35 ± 5.23 vs 82.28 ± 5.19; *P* = 0.942). The HRs at T_2_, T_3_, T_4_, and T_5_ in EXG were markedly lower than those in COG (62.38 ± 5.19 vs 68.33 ± 5.27, 66.30 ± 5.18 vs 95.27 ± 5.23, 67.30 ± 5.24 vs 97.33 ± 5.16, and 65.40 ± 5.27 vs 88.28 ± 5.16; *P* < 0.001).

No obvious differences were shown in MAP at T_1_ between EXG and COG (70.30 ± 5.26 vs 70.35 ± 5.22, *P* = 0.958). The MAP at T_2_, T_3_, T_4_, and T_5_ in EXG was markedly lower than that in COG (76.32 ± 5.20 vs 80.30 ± 5.18, 85.25 ± 5.23 vs 92.28 ± 5.20, 68.33 ± 5.23 vs 75.30 ± 5.24, and 65.32 ± 5.16 vs 72.33 ± 5.21; *P* < 0.001).

### 3.2. Comparison of Stress Indexes

**Figure 2:**
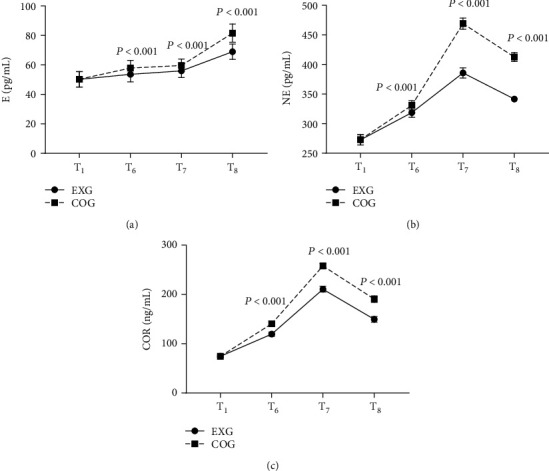
Comparison of stress indexes ($\bar{x}\pm s$). (a) Showed epinephrine (E) levels (pg/ml); (b) showed norepinephrine (NE) levels (pg/mL); (c) showed cortisol (COR) levels (ng/ml).

The stress indexes at T_6_, T_7_, and T_8_ were notably lower in EXG than in COG (*P* < 0.001), as shown in Figure 2.

No obvious differences were shown in the E levels at T_1_ between EXG and COG (50.21 ± 5.22 vs 50.29 ± 5.24, *P* = 0.933). The E levels at T_6_, T_7_, and T_8_ in EXG were markedly lower than those in COG (53.66 ± 5.10 vs 57.88 ± 5.20, 55.98 ± 4.50 vs 59.65 ± 4.27, and 68.98 ± 5.16 vs 81.55 ± 6.21; *P* < 0.001).

No obvious differences were shown in the NE levels at T_1_ between EXG and COG (272.65 ± 8.55 vs 273.10 ± 8.47; *P* = 0.773). The NE levels at T_6_, T_7_, and T_8_ in EXG were markedly lower than those in COG (318.65 ± 7.88 vs 330.98 ± 7.80, 385.65 ± 8.54 vs 468.98 ± 9.41, and 341.65 ± 4.77 vs 412.58 ± 7.52; *P* < 0.001).

No obvious differences were shown in the COR levels at T_1_ between EXG and COG (74.55 ± 2.65 vs 74.58 ± 2.47; *P* = 0.949). The COR levels at T_6_, T_7_, and T_8_ in EXG were markedly lower than those in COG (119.65 ± 4.21 vs 140.65 ± 5.47, 210.65 ± 5.88 vs 257.98 ± 4.56, and 149.65 ± 5.74 vs 190.65 ± 6.41; *P* < 0.001).

### 3.3. Comparison of Postoperative Pain Scores

The pain scores at T_6_, T_7_, and T_8_ in EXG were markedly lower than those in COG (1.70 ± 0.53 vs 3.32 ± 0.50, 1.63 ± 0.48 vs 2.67 ± 0.51, and 1.47 ± 0.53 vs 1.93 ± 0.48; *P* < 0.001).

### 3.4. Comparison of Incidence of Adverse Reactions

The incidence of dizziness, headache, pruritus, and emergence agitation in EXG was lower compared with that in COG (*P* < 0.05), as demonstrated in Table 2.

## 4. Discussion

Due to the short duration of radical mastectomy and low requirements for muscle relaxation, fast-track anesthesia is often applied in clinic, such as total intravenous anesthesia (TIVA) [14]. TIVA refers to the general anesthesia by intravenous injection of anesthetic drugs that act on the central nervous system through blood circulation. In order to ensure the stability of anesthesia and alleviate physiological disturbance, TIVA is generally achieved by a combination of drugs. However, the efficacy of the combined drugs cannot be accurately predicted because the combination may have synergistic and additive effects as well as antagonistic effects, leading to pharmacodynamic changes. Therefore, the compound effects of different drugs need to be analyzed one by one to clarify the drug efficacy of their combination [15–17]. This study combined two drugs (fentanyl and sufentanil) with propofol. Since fentanyl is rarely involved in previous studies, there is a lack of a large number of control studies to evaluate its application effect. Zhang et al. [18] believed that there was a synergistic effect between fentanyl and propofol. From their point of view, when propofol was used alone, 3.3 *μ*g/ml and 27.4 *μ*g/ml of steady-state blood concentrations were required to make 50% of patients unresponsive to oral commands and skin incision, while 3.26 ng/ml and 4.17 ng/ml of steady-state blood concentrations were required for patients without movement and endocrine disorders when propofol combined with fentanyl was used. This shows that the combination of fentanyl and propofol can improve the analgesic and sedative effects. However, excessive application of fentanyl can lead to reactions such as drowsiness, nausea, and vomiting, and even death due to respiratory depression in severe cases [19, 20]. Therefore, dosage control of fentanyl is required, which has always been the key and difficult content of TIVA.

Compared with fentanyl, sufentanil has faster clearance and does not accumulate in patients due to its smaller volume of distribution, shorter half-life, and shorter terminal clearance period, with fewer postoperative adverse reactions, so it is easier to achieve the controllable amount of anesthesia [21, 22]. Moreover, sufentanil is a thienyl derivative at the N-4 position of fentanyl and its receptor affinity is about 7–10 times that of fentanyl. Therefore, sufentanil has a stronger inhibitory effect on the stress response induced by noxious stimuli [7]. The clinical manifestations of stress response include increased blood pressure, accelerated heart rate (HR), and increased cardiac oxygen consumption. Therefore, the HR and MAP in EXG were more stable and the stress response indexes at T_6_, T_7_, and T_8_ were lower in EXG than in COG (*P* < 0.001). It is worth noting that the mechanism of sufentanil stabilizing HR is to stabilize peripheral vascular resistance, excite the central vagus nerve nucleus, and block the sympathetic nerve. Therefore, patients with bradycardia should be closely watched and treated with atropine if necessary [23].

The analysis by Seokha et al. [24] showed that sufentanil, the most effective fentanyl family drug at present, could not only maintain intraoperative analgesia, but also have good postoperative analgesic effects. The application of sufentanil combined with propofol for TIVA in laparoscopic appendectomy prolongs the analgesic time and has better effects, especially for short-term postoperative analgesia and sedation, with greater postoperative comfort for patients. This study found that the postoperative pain scores at T_6_, T_7_ and T_8_ in EXG were lower compared with COG (P <0.001), which was consistent with the study of Seokha et al. In addition, clinical reports have shown that adverse drug reactions of sufentanil are mostly the same as those of fentanyl, mainly including respiratory depression, skeletal muscle stiffness, tachycardia, arrhythmia, and pruritus. With no respiratory depression in this study, the main adverse reactions of patients included nausea and vomiting, dizziness and headache, and abdominal distension. The incidence of dizziness, headache, pruritus, and emergence agitation in EXG was lower compared with that in COG (*P* < 0.05), suggesting that sufentanil had faster clearance and better safety, which is conducive to the better quality of the recovery period.

In conclusion, sufentanil combined with propofol for TIVA can stabilize intraoperative hemodynamic indices of patients undergoing radical mastectomy, alleviate perioperative stress response, and reduce pain perception. Therefore, this anesthesia method is safe and merits clinical promotion.

## Figures and Tables

**Table 1:** Comparison of clinical data between the two groups.

Items	EXG (*n* = 60)	COG (*n* = 60)	*X* ^2^/*t*	*P*
Average age ($\bar{x}\pm s$, yrs)	52.28 ± 5.20	52.32 ± 5.21	0.042	0.967
Body weight ($\bar{x}\pm s$, kg)	62.65 ± 3.21	62.87 ± 3.20	0.376	0.708
BMI ($\bar{x}\pm s$, kg/m^2^)	22.35 ± 1.21	22.41 ± 1.23	0.269	0.788
ASA classification			0.135	0.714
I	32	34		
II	28	26		

BMI: body mass index; ASA: American Society of Anesthesiologists.

**Table 2:** Comparison of incidence of adverse reactions [*n*(%)].

Group	*n*	Nausea and vomiting	Dizziness and headache	Drowsiness	Palpitation	Intolerance of cold	Pruritus	Abdominal distension	Emergence agitation
EXG	60	4(6.7)	1(1.7)	3(5.0)	1(1.7)	1(1.7)	0(0.0)	2(3.3)	0(0.0)
COG	60	9(15.0)	8(13.3)	3(5.0)	2(3.3)	1(1.7)	4(6.7)	6(10.0)	10(11.7)
*X* ^2^		2.157	5.886	0.000	0.342	0.000	4.138	2.143	10.909
*P*		0.142	0.015	1.000	0.559	1.000	0.042	0.143	0.001

## Data Availability

Data to support the findings of this study is available upon reasonable request from the corresponding author.
